# Mothers of small-bodied children and fathers of vigorous sons live longer

**DOI:** 10.3389/fpubh.2023.1057146

**Published:** 2023-01-25

**Authors:** Markus Valge, Richard Meitern, Peeter Hõrak

**Affiliations:** Department of Zoology, University of Tartu, Tartu, Estonia

**Keywords:** anthropometric traits, body size, inter-generational study, longevity, obesity, sex difference

## Abstract

Life-history traits (traits directly related to survival and reproduction) co-evolve and materialize through physiology and behavior. Accordingly, lifespan can be hypothesized as a potentially informative marker of life-history speed that subsumes the impact of diverse morphometric and behavioral traits. We examined associations between parental longevity and various anthropometric traits in a sample of 4,000–11,000 Estonian children in the middle of the 20th century. The offspring phenotype was used as a proxy measure of parental genotype, so that covariation between offspring traits and parental longevity (defined as belonging to the 90th percentile of lifespan) could be used to characterize the aggregation between longevity and anthropometric traits. We predicted that larger linear dimensions of offspring associate with increased parental longevity and that testosterone-dependent traits associate with reduced paternal longevity. Twelve of 16 offspring traits were associated with mothers' longevity, while three traits (rate of sexual maturation of daughters and grip strength and lung capacity of sons) robustly predicted fathers' longevity. Contrary to predictions, mothers of children with small bodily dimensions lived longer, and paternal longevity was not linearly associated with their children's body size (or testosterone-related traits). Our study thus failed to find evidence that high somatic investment into brain and body growth clusters with a long lifespan across generations, and/or that such associations can be detected on the basis of inter-generational phenotypic correlations.

## 1. Introduction

### 1.1. Background: Coevolution and clustering of life-history and anthropometric traits

According to the theory of life-history evolution, the key traits that contribute to Darwinian fitness, i.e., growth, survival and reproduction, evolve in a coordinated manner. The theory views the evolution of these traits as the product of interactions between intrinsic constraints and trade-offs as well as extrinsic factors in the environment that affect mortality risk and resource availability (1–3). Trade-offs represent the costs paid in fitness when a beneficial change in one trait is linked to a detrimental change in another. Microevolutionary trade-offs reflect negative genetic correlations between fitness-enhancing traits (4, 5). Physiological trade-offs manifest at the level of phenotype (e.g., growth stunting in response to infections that require allocating somatic investments into immune responses at the expense of growth).

Life-history traits (traits directly related to survival and reproduction) materialize through physiology and behavior. This means that in addition to basic components of fitness, such as fecundity and age-specific survival probability, values of anatomical, physiological, behavioral and psychosocial traits too accumulate non-randomly among the individuals within populations (2, 3, 6, 7). This premise is well-supported by genome-wide association (GWA) studies demonstrating moderate to strong genetic correlations between life-history and associated physiological, behavioral and health-related traits (8–11).

Life-history (and related) traits co-evolve because they adapt to the same environment. For example, low levels of extrinsic and intrinsic mortality typically favor coevolution and genetic clustering of qualities characteristic to slow pace of life—including slow maturation and high somatic investment into body and brain growth, delayed reproduction, long lifespan, conscientious personality and propensity for relatively high parenting effort in relation to mating effort (7, 12, 13) [Note, however, that evidence for a single fast-slow continuum of the pace of life in humans is mixed, as discussed by Sear (14)].

### 1.2. Lifespan as a potentially informative marker of life-history speed

Age at death in humans is heritable [maximum *h*^2^ = 0.25–0.4; reviewed by (15–17)] with genetic influence increasing with age (18, 19). Also, longevity (defined here as belonging to the 90th percentile of the lifespan of the population) correlates genetically with multiple diseases and traits, such as coronary artery disease (*r*_g_ = −0.40), age of first birth (*r*_g_ = 0.33) and years of schooling (*r*_g_ = 0.26) (20). With the epidemiologic transition in the middle of the 20th century, non-communicable diseases have become the major causes of death in the developed world, with behavioral (including lifestyle and dietary) risk factors largely responsible for the variation in the lifespan (21). Psychometric traits relating to such behaviors include impulsivity, sensation-seeking and delay discounting, all of which show moderate to high heritabilities [reviewed by (22)]. A recent piece of evidence for coevolution of behavioral traits and longevity comes from a UK biobank study, showing that genetic variants associated with delayed age of first sexual intercourse predicted subjects' chances of becoming longevous better than most of polygenic scores for disease or physical measures (23).

Lifespan can thus be considered a potentially informative marker of life-history speed that subsumes the impact of many anthropometric (including behavioral) traits. However, measuring phenotypic correlations between lifespan vs. other traits of interest at the level of individuals (though interesting on its own merit) does not enable to distinguish between phenotypic and microevolutionary trade-offs. In intergenerational studies, this problem can be indirectly circumvented by taking advantage of the fact that children inherit 50% of each of their parents' genomes so that the phenotype (24–26) or genotype (20, 27, 28) of offspring is used as a proxy measure of parental genotype [see (29)]. For instance, measuring the body mass index (BMI) in the offspring and age at death of their parents enables to exclude some of the potentially confounding effects of external factors such as ill health that could affect the BMI and lifespan simultaneously when both are measured in the same individual (24). As an example of the utility of such an approach, it was shown recently that parents of early maturing girls die younger, a finding that strongly supports the existence of microevolutionary trade-offs between two central life-history traits (22).

### 1.3. Previous intergenerational studies on offspring phenotype vs. parental age at death

So far, few studies have used offspring morphometric traits to predict parental longevity and lifespan, and all of these were based on parent-son comparisons. In a study of an entire community of Tecumseh, Michigan, the longevity of either parent was related to high values of ventilatory lung function among the sons (30). A study of over a million Swedish conscripts established that parents of taller sons had lower mortality overall and from cardiovascular and coronary heart diseases, stroke, diabetes, respiratory disease, external causes and suicide, while sons' height was positively associated in both parents with mortality from cancer (26). Two other studies of the same sample found that sons' BMI was positively associated with all-cause and cardiovascular mortality of their parents (25, 29).

Genome-wide association study in the UK Biobank (UKBB) that examined parental survival in relation to offspring genotype (pooled over sexes of offspring and parents) demonstrated positive genetic correlations between childhood obesity (*r*_g_ = 0.30), childhood height (*r*_g_ = 0.12), adult hip circumference (*r*_g_ = 0.28) and parental mortality. At the same time, birth weight was negatively associated with parental mortality (*r*_g_ = −0.21) (31). Another study in the UKBB showed positive genetic correlations in the magnitude of 0.34 and 0.39 between the polygenic score of handgrip strength vs. parents' and fathers' age at death, respectively (32). Height-increasing polygenic scores were inversely associated with extreme longevity in Japanese women but not men (33).

### 1.4. Study aims in the context of opposing scenarios of selection

Here we examine associations between parental longevity and 16 anthropometric traits of their children, measured at school age. The dataset collected by Prof. Juhan Aul in the middle of 20th century Estonia [see (34, 35)] is particularly suitable for the study of longevity because 93–98% of participants' parents were dead, which results in high statistical power to detect associations between offspring traits and parental longevity [e.g., (36)]. Following (18, 20, 23), we concentrate on the analysis of longevity, comparing the phenotypes of offspring of parents that belong to the 90th survival percentile (mothers 92 years and older, fathers 88 years and older) with those of parents whose age at death was at or below the 60th survival percentile [mothers 85 years and younger, fathers 78 years and younger (see Methods for explaining the rationale of such an approach)].

As regards general predictions about the association between offspring phenotype and parental longevity, opposing scenarios can be imagined. On the one hand, smaller individuals can be predicted to live longer than larger ones because of decreased cancer risk due to a smaller number of cells whose replication involves the risk of DNA damage (26). In addition, growing, birthing, and breastfeeding larger babies could negatively impact mothers' health and lifespan (37). On the other hand, taller individuals have a number of health advantages that stem from their better educational attainment and cognitive abilities (38), greater lung capacity [protective against respiratory (39) and cardiovascular (40) disease] and higher birth weights (41). However, such associations might be at least partly explained by residual confounding, for example, by socio-economic position (SEP) or confounding due to existing but undiagnosed illness, sometimes also referred to as reverse causality [reviewed by (26)].

### 1.5. Anthropometric traits

Studied traits include leg length [a sensitive marker of pre-pubertal growth conditions (42) and metabolic and reproductive health (43)] and various measures of the face and body size and shape that are associated with perceived masculinity and testosterone exposure (42). Among these, handgrip strength and lung capacity are indices of physical ability and robustness, predicting short- and long-term morbidity and mortality in adult populations (44). In our study population, childhood handgrip strength predicted the reproductive success of men (but not women) (45), while cranial volume predicted educational attainment in both sexes (46).

### 1.6. Predictions

All the linear size measures in our study are positively correlated with each other and with indices of physical ability (35, 46). Assuming that these indices, measured in children, partly reflect the genotypes of their parents, we predicted that (i) parents of robust children with larger linear dimensions have higher chances of becoming longevous. Additionally, we predicted that (ii) parents of children with large crania live longer given that brain and cranial volumes correlate (both phenotypically and genetically) with intelligence and educational attainment (46, 47), which, in turn, predict longer life [reviewed by (48)]. Because a sizeable proportion of men die from external causes that associate with testosterone-dependent behavior, we also predicted that (iii) anthropometric traits that reflect testosterone exposure (and/or amount in circulation) such as face and jaw width, face roundness, shoulder width and shoulder/hip ratio [see (46)] associate positively with paternal mortality. Finally, we measured the associations between the breast development rate of daughters (a marker of the speed of sexual maturation) and parental longevity. We previously established that this trait associates positively with parental mortality in the survival analyses (22), so we predicted that (iv) it would also show negative associations with longevity.

## 2. Methods

### 2.1. Data collection and study sample

Data on morphometric measurements and family background were obtained from the anthropometric study performed by Juhan Aul between 1956 and 1969 [for the historical background of this sample, see (34, 49)]. Details for measurements are described in (35): Hip width (bicristal diameter) was measured as the distance between the external margins of the iliac crests. Shoulder width (biacromial diameter) was measured as the horizontal distance across the shoulders measured between the acromia. Cranial volume was estimated according to the formula 7.884^*^(head length−11) + (10.842^*^head width−11) – 1593.96 (units in mm). Face roundness (lfWHR) denotes lower face width/height ratio. Vital capacity of lungs was measured using a bellows-type spirometer. Maximum handgrip strength was registered with a handheld dynamometer. The development stage of breasts was assessed on the basis of the six-point Tanner scale (0–5). All measurements were recorded by a single person.

Age- and sex-specific residuals of anthropometric traits were calculated from generalized additive models in which the focal trait was regressed against smooth non-parametric functions of age (in days) and birth date using package “gam” for R (50) and [(R syntax: focal_trait ~ s(age) + s(birth_date)]. We included birth date as a predictor to account for the steady increase in age-adjusted body dimensions over the study period [see (34)]. Residuals were then standardized to z-scores within the sexes. All results presented here are based on these standardized residuals rather than raw trait values. The full dataset for calculation of residuals of morphometric traits consisted of 9,586–15,253 girls (depending on the trait) and 7,170–11,842 boys [age range 6.4–20.0 years, mean = 12.7 ± 6.4 (SD) years, born between 1937 and 1962]. Given the correlations between traits, we also performed principal component (PC) analysis as described in (45). All linear measures and handgrip strength loaded positively to PC1 (termed hereafter as size) and explained altogether 81% of its variance.

From the total sample, we identified individuals whose fathers (*n* = 11,502) and/or mothers (*n* = 11,244) age of death (or age, if alive) was recorded in the Estonian Population Registry as at 2018. Sample sizes vary between analyses because participants differ with respect to the number of anthropometric and biosocial traits recorded. To eliminate reverse causation (i.e., the effect of parental absence on the growth of their children [see (42)], all participants whose mother and/or father had died before measuring were excluded from the analysis.

### 2.2. Division of sample to 60th and 90th longevity percentiles

To analyse the association between anthropometric traits of children and the longevity of their parents, we proceeded from the method of (18) and (20), comparing the phenotypes of offspring of parents that belong to the 90th survival percentile (mothers 92 years and older, fathers 88 years and older) with those of parents whose age at death was at or below the 60th survival percentile (mothers 85 years and younger, fathers 78 years and younger). In addition, we added to the 90th percentile 179 fathers and 759 mothers born before 1928 and still alive in 2018. The rationale for excluding parents whose age at death fell between 60th and 90th survival percentile is that the heritable component of longevity is strongest in individuals belonging to the top 10% survivors of their birth cohort (18) and defining longevity on the basis of top 10% survivors is becoming normative approach in aging studies (20, 23).

### 2.3. Data analysis: Stratification, confounders, and mediators

We analyse the association between offspring phenotype and parental longevity/mortality among the sample of children pooled over sexes and also for sons and daughters separately because pedigree studies have shown that inheritance patterns of longevity/lifespan may depend on the sex of parents as well as their offspring [reviewed in (15)], likely because different pathways contribute to longevity in men and women (51). To control for potentially confounding effects of social origin and cost of reproduction on parental longevity [e.g., (52)], we adjust our analyses for the parental socio-economic position, urban vs. rural origin and number of children. Because the theory of life-history evolution predicts that reproductive effort is costly in terms of future survival (1, 53), we start with examination of the relationship between parental longevity and the number of their offspring.

### 2.4. Statistics

The odds of becoming longevous (i.e., belonging into the 90th vs. 60th longevity percentile were analyzed with logistic regression using package “rms” for R (54). The non-linear associations between offspring traits and parental longevity were analyzed by testing the significance of squared trait value in the model.

**Figure 1:**
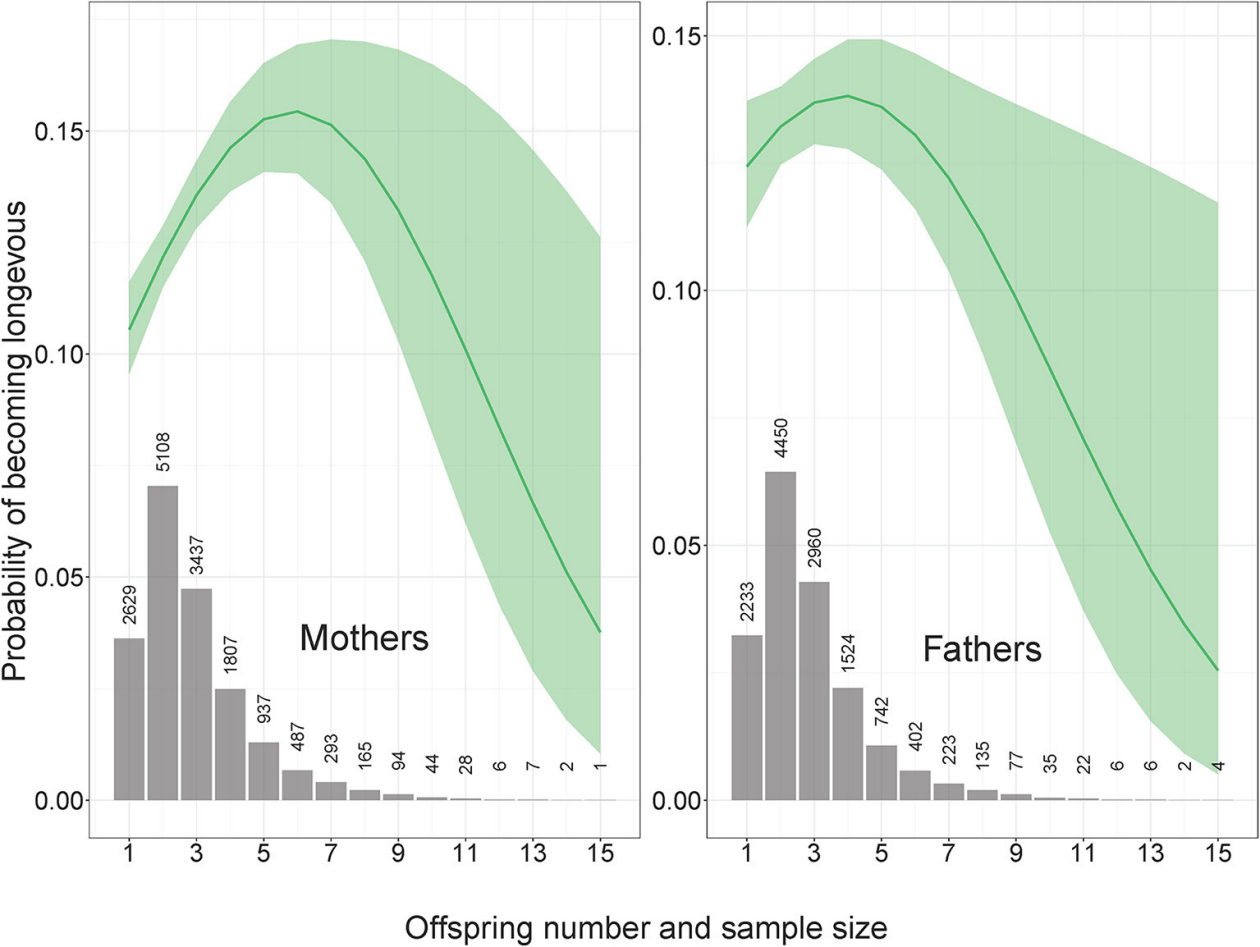
Probability of becoming longevous as a function of offspring number. Mothers: OR (95% CI) for offspring number and offspring number squared 1.24 (1.12–1.37) and 0.98 (0.97–0.99), n longevous = 1,477, n non-longevous = 9,990. Fathers: OR (95% CI) for offspring number and offspring number squared 1.12 (1.00–1.25) and 0.99 (0.97–1.00), n longevous = 1,273, n non-longevous = 8,383. In all figures OR denotes odds ratio and CI means confidence intervals.

All logistic regression models were adjusted for parents' year of birth. Additionally, models for mothers were adjusted for rural vs. urban origin and a binary factor of SEP (unskilled manual workers vs. skilled professions). Models for fathers were adjusted for the three-level factor of SEP (1: unskilled manual, 2: skilled manual, and 3: non-manual professions). For instance, the R syntax for the model testing whether paternal odds of becoming longevous depended on the height and its squared value [adjusting for fathers' year of birth and socioeconomic position was as follows: lrm(formula = father_longevous ~ father_year_of_birth + father_socioeconomic_position + son_height + son_height ∧ 2, data = data)]. Additionally, we run all the models with parental offspring number and its squared value to test for the possibility that the curvilinear association between offspring number and parental longevity (Figure 1) would affect the association between anthropometric traits of children and the longevity of their parents. Confidence intervals that do not include one are considered statistically significant at the 95% level. The dataset's descriptive statistics are presented in Table 1.

**Table 1:** Descriptive statistics for parents' birth years and ages at death in a sample used for analyzing association between offspring anthropometric traits and parental longevity.

**Mothers' SEP**	**Origin**	**Mother's birth year**						**Mother's age at death**	
		**Mother alive**			**Mother dead**					
		***n***	**Mean (SD)**	**Range**	***n***	**Mean (SD)**	**Range**	**Mean (SD)**	**Range**
1	Rural	134	1927.9 (4.1)	1,918–37	3,415	1917.5 (7.5)	1,887–1,937	78.3 (11.0)	36–105
2	Rural	67	1928.1 (3.7)	1,921–33	695	1921.7 (6.6)	1,897–1,941	79.2 (11.1)	34–102
1	Urban	233	1929.9 (4.7)	1,919–41	3692	1919.0 (8.0)	1,890–1,942	78.7 (11.0)	37–103
2	Urban	325	1930.2 (4.4)	1,916–42	2681	1921.8 (7.4)	1,885–1,942	78.5 (11.3)	35–106
**Fathers' SEP**		**Father's birth year**						**Father's age at death**	
		**Father alive**			**Father dead**				
		***n***	**Mean (SD)**	**Range**	***n***	**Mean (SD)**	**Range**	**Mean (SD)**	**Range**
1		52	1928.6 (4.4)	1,921–42	6,099	1914.1 (9.3)	1,876–1,940	72.6 (12.0)	22–104
2		61	1931.0 (5.3)	1,921–44	2,499	1918.4 (8.3)	1,886–1,942	73.0 (12.0)	25–103
3		66	1929.0 (4.2)	1,921–41	1,961	1917.3 (8.6)	1,885–1,937	74.2 (12.0)	23–103

Mothers' Socioeconomic Position (SEP): Unskilled manual workers (1), skilled professions (2). Fathers' SEP: Unskilled manual (1), skilled manual (2) and non-manual (3) workers.

Data processing was performed anonymously under the license of the Research Ethics Committee of the University of Tartu (protocol # 275/ T-1, issued on 20.11.1017) and approved by the Estonian Data Protection Directorate (Decision n2 2.2.-1/17/55, issued on 30.01.2018).

## 3. Results

### 3.1. Offspring traits vs. maternal longevity

**Figure 2:**
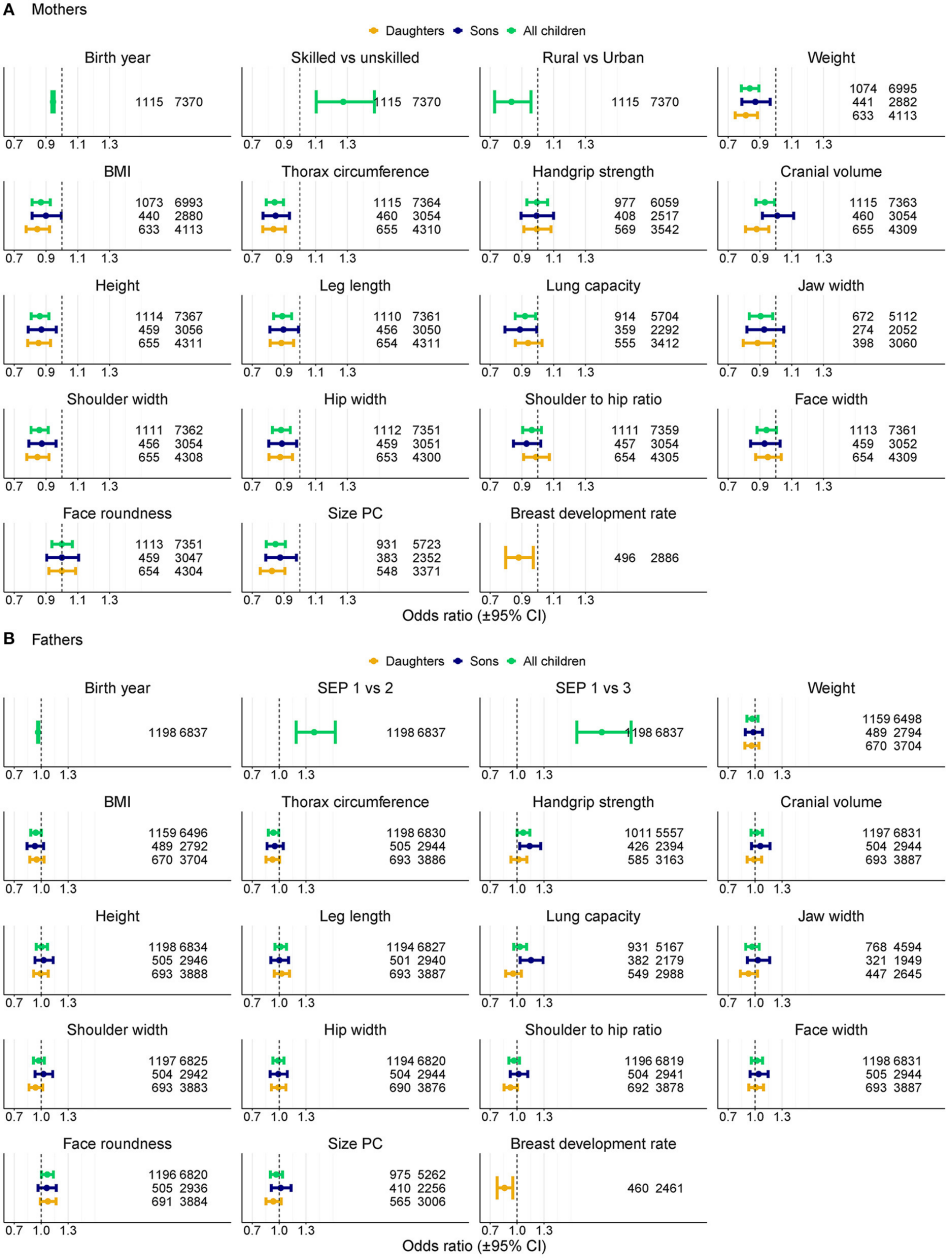
Association between anthropometric traits of children and longevity (i.e., odds of belonging to the top 90th percentile vs. below 60th percentile of lifespan) of their parents in logistic regression models. ORs for anthropometric traits are per one standard deviation of trait z-score values. OR for year of birth is per year, Rural is for rural vs. urban origin. SEP for mothers is skilled professions vs. manual unskilled workers, SEP for fathers is 1 for unskilled manual, 2 for skilled manual and 3 for non-manual professions. Models for all anthropometric traits control for parents' year of birth, SEP and rural origin (for mothers only). Numbers denote sample sizes for longevous vs. non-longevous parents. **(A)** Mothers, **(B)** Fathers.

**Figure 3:**
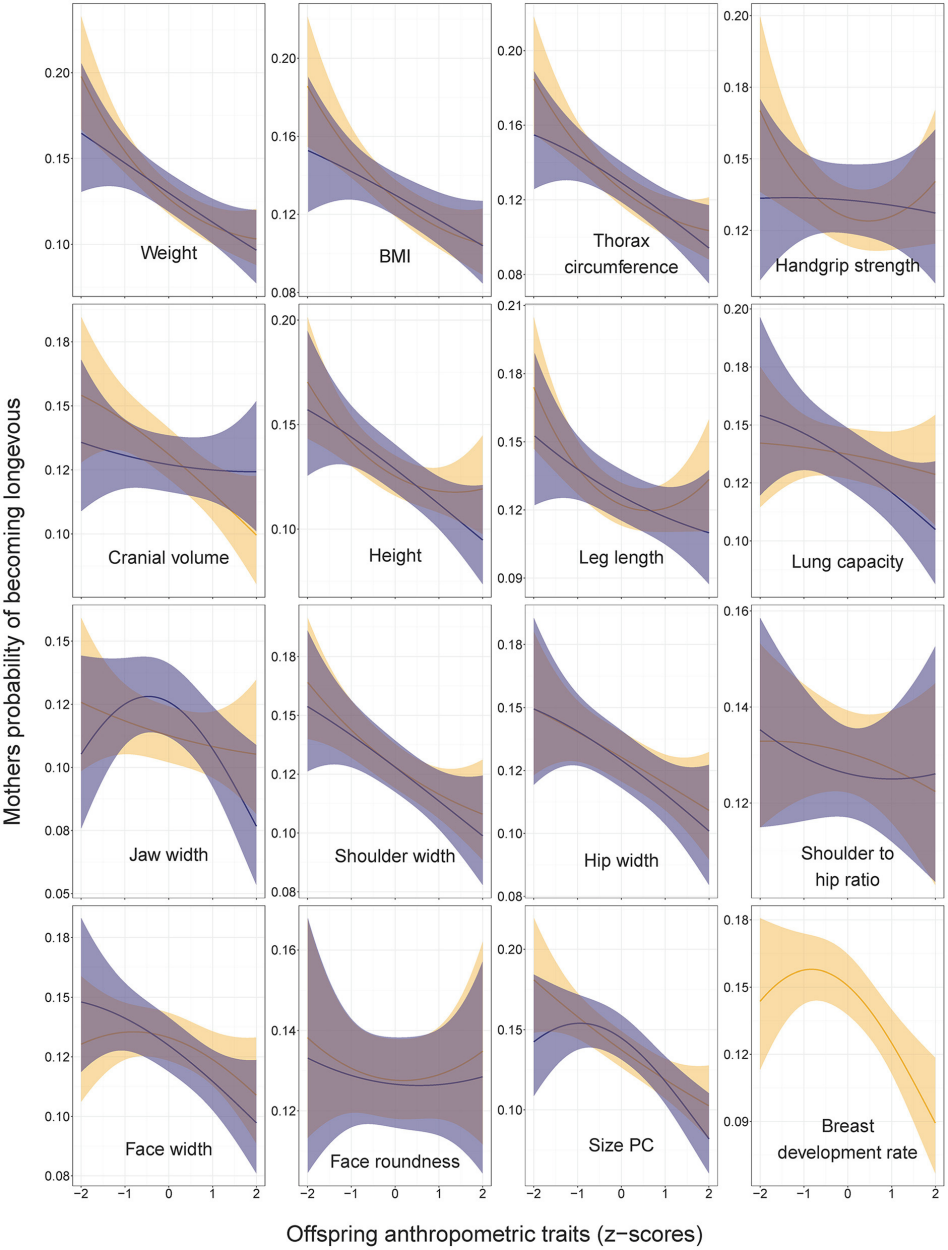
Mothers' probabilities of becoming longevous in relation to anthropometric traits of their sons (dark shading) and daughters (light shading). Predictions (with 95% CI) from the models without covariates: lrm[formula = longevity ~ focal_trait + (focal_trait*focal_trait)]. Statistics are provided in Supplementary Table 1 in ESM.

The number of children was associated with the maternal probability of becoming longevous in a non-linear way, increasing with the number of children from one to six and decreasing with further increases in parity (Figure 1). Among the mothers of participants, odds of surviving to 90th percentile were most strongly related to SEP and urban vs. rural origin (Figure 2A, Supplementary Table 1 in ESM). Women in skilled professions had 27% higher odds of becoming longevous than women in unskilled manual professions. Women in rural areas had 17% lower odds of becoming longevous than women in urban areas. Twelve of 16 anthropometric traits of offspring were associated with the longevity of their mothers (Figures 2A, 3, Supplementary Table 1 in ESM): when sons and daughters were pooled for the analysis, children of longevous mothers were generally smaller than children of the mothers whose age at death was at or below the 60th survival percentile. They were shorter, had shorter legs, narrower shoulders and jaws, and smaller thorax circumference. They also weighed less and had smaller BMI and lung capacity and slower rates of sexual maturation, assessed on the basis of breast development rate. Cranial volume had sex-specific association with maternal longevity, as indicated by significant interaction term with offspring sex [standardized regression coefficient (β) for interaction = −0.13, SE = 0.07, *P* = 0.040; Supplementary Table 3 in ESM]. Mothers of daughters (but not sons) with larger crania had lower odds of becoming longevous. Strongest predictors of maternal longevity were daughters' weight (OR = 0.81, CI = 0.74–0.88) and BMI (OR = 0.85, CI = 0.77–0.92). That is, an increase of daughters' weight by one SD reduced their mothers' chances to become longevous by 19%, an effect on the magnitude of living in a rural area.

Non-linear associations between measures of offspring body size and mothers' longevity were detected for two traits. Mothers' odds of becoming longevous decreased with the leg length of their daughters in a non-linear, U-shaped manner (Figure 3; Supplementary Table 1 in ESM: OR for leg length squared = 1.06, CI = 1.00–1.12). An inverse J-shaped relationship was observed between mothers' odds of becoming longevous and the jaw width of their sons (Figure 3; Supplementary Table 1: OR for jaw width squared = 0.90, CI = 0.82–0.99). Similar pattern was observed for the size PC (Figure 3; Supplementary Table 1 in ESM: OR for PC1 squared = 0.91, CI = 0.83–0.99). Associations between anthropometric traits of offspring and longevity of their mothers remained virtually identical irrespective of the inclusion of the mothers' number of children and its squared value in the models (Supplementary Table 2 in ESM).

### 3.2. Offspring traits vs. paternal longevity

The number of children was associated with paternal probability of becoming longevous in a non-linear way, increasing with the number of children from one to four and decreasing with further increases in parity (Figure 1). Longevity of the fathers of participants was most strongly associated with their SEP. Men in non-manual professions had 1.9 times, and men in skilled manual professions had 1.4 times higher chances of becoming longevous than men in unskilled manual professions (Figure 2B, Supplementary Table 1 in ESM) while rural/urban residence was not associated with longevity in a model accounting for SEP. Fathers' longevity was independent of their children's anthropometric traits when their sons and daughters were pooled for the analysis (Supplementary Table 1 in ESM). The single trait of daughters that predicted longevity of their fathers was breast development rate: men whose daughters exhibited a slower sexual maturation rate had higher chances of becoming longevous (OR = 0.86, CI = 0.78–0.95; Figure 3). Among sons, only traits associated with physical ability/vigor predicted parental longevity: fathers of stronger sons with higher lung capacity had higher odds of becoming longevous (OR = 1.14, CI = 1.03–1.26 and OR = 1.15, CI = 1.03–1.29 for grip strength and lung capacity, respectively; Figure 3). The latter association was opposite to that observed in mothers. Lung capacity also had sex-specific association with paternal longevity, as indicated by significant interaction term with offspring sex (β for interaction = −0.19, SE = 0.07, *P* = 0.008; Supplementary Table 3 in ESM).

Three traits showed bell-shaped associations with fathers' odds of becoming longevous (Figure 3). Fathers of sons with average dimensions of leg length, height and size PC had higher chances of becoming longevous than fathers of children with extreme values of these traits (OR for sons' leg length squared = 0.90, CI = 0.83–0.97; OR for sons' height squared = 0.93, CI = 0.86–1.00; OR for sons' PC1 squared = 0.88, CI = 0.81–0.97). Associations between anthropometric traits of offspring and longevity of their fathers remained practically identical irrespective of the inclusion of the fathers' number of children and its squared value in the models (Supplementary Table 2 in ESM).

## 4. Discussion

### 4.1. Differences in associations between anthropometric traits of children and longevity of their mothers and fathers

The most prominent finding of this study is that many anthropometric traits of offspring were linearly associated with longevity of their mothers, while only three offspring traits (rate of sexual maturation of daughters and grip strength and lung capacity of sons) predicted longevity of fathers in a straightforward way. One explanation might stem from the sex difference in lifespan in Estonia, which has been among the largest in Europe since at least 1980 [along with Latvia and Lithuania, (55)]. Unlike Western Europe, Estonia's life expectancy stagnated between 1960 and 1995 (56). Despite increasing life expectancy since 1995, the male disadvantage of 9–10 years in Estonia has remained unchanged from 1976 to 2016, peaking at 12.3 years in 1994 (57). This means that the bulk of mortality of fathers and mothers of the participants of our study occurred in different periods: 50% of fathers (interquartile range) in our study died between 1980 and 1999, while the corresponding period for mothers occurred later, from 1989 to 2007.

Since from the mid-1990s, mortality rates from treatable causes (mainly diseases of the circulatory system and some curable cancers) have been decreasing in Estonia (58), it is possible that mothers of our participants had a lower chance of dying prematurely due to deficiencies in health care and public health policies than fathers. In the context of our findings that many more offspring traits were associated with longevity of mothers than fathers and that fathers were more likely to die of preventable causes (see below), one might thus speculate that anthropometric traits of offspring predict better “timely” than premature mortality.

Another, mutually not exclusive explanation for why offspring traits predicted longevity better in the case of mothers than fathers might relate to maternal effects. For instance, maternal hyperglycaemia during pregnancy has been associated with increased offspring adiposity in childhood (59). Under this scenario, diabetic mothers (with reduced life expectancy) are more likely to have obese children. Such a scenario might explain why we observed relatively strong associations between the weight of children and the longevity of their mothers while the weight of children was unrelated to the longevity of their fathers. Such reasoning, however, fails to explain why mothers (but not fathers) of children with shorter stature and legs and narrower shoulders had high chances of becoming longevous.

A possible explanation of why mothers but not fathers of small-bodied children became longevous might be the sex-specific cost of reproduction, i.e., energetic and somatic costs incurred throughout pregnancy and lactation of large-bodied babies (37). Note, however, that such an explanation would mean that offspring size is more important in terms of shortening maternal lifespan than offspring number, as for the bulk of the population, maternal chances of becoming longevous increased with offspring number (Figure 1). Similar patterns where the mortality hazard rate decreases as parity increases up to 3 children were observed in a meta-analysis of 37 studies from samples of developed nations that were gathered after 1945 [(60); see also (36)]. It should be noted that in the current study (like in many others), the pattern of association between offspring number and parental mortality was generally similar for mothers and fathers (Figure 1). This finding is difficult to reconcile with the idea that mothers of large-bodied girls had smaller chances of becoming longevous due to energetic and somatic costs of birthing and nursing large infants. If the relationship between human fertility and longevity is dominated by physiological trade-offs, one should expect significantly higher costs of reproduction for women than for men (61).

It may also be possible that children are more similar to their mothers than to fathers with respect to anthropometric traits (e.g., due to a greater degree of shared environment or mis-assigned paternity) or that the associations between mortality and own anthropometric traits are stronger among women than among men (26). Inheritance of lifespan may be also higher in the maternal than paternal line (15).

Yet another explanation for the sex differences of associations between anthropometric traits of children and longevity of their parents might stem from different causes of death between the mothers and fathers of participants. During the study period, the three most common causes of death in Estonia were diseases of the circulatory system, neoplasms, and external causes, i.e., deaths due to injuries and poisoning. Diseases of the circulatory system were more common among women than men (73 vs. 56%, respectively; averages from 1989 to 2018; Supplementary Figure S1 in Supplementary Table 5 in ESM). The proportion of deaths due to neoplasms was almost similar among women and men (22 and 24%), while deaths due to external causes dominated among men (18 vs. 5%). It may thus appear that smaller body dimensions provide particular survival advances in the case of diseases of the circulatory system (that relate to conditions associated with metabolic syndrome) and because women more often than men die due to such diseases, sex differences in associations between offspring phenotype and parental mortality emerged. Additionally, it is possible that deaths due to external causes (that are far more prevalent among men) are less strongly associated with offspring phenotypes than other causes of death.

Notably, the latter explanation would be difficult to reconcile with the concept that testosterone-dependent traits associate with risk-prone behavior (62, 63) and steeper discounting of the future (64). More generally, the theory of sexual selection predicts that males pursue a “live fast, die young” life history pattern due to the increased risk of extrinsic mortality associated with obtaining mates (65, 66) and the main mechanism for this is mediated by the adverse effects of testosterone on their physiology and behavior (63, 67). Contrary to these predictions, we saw that mothers (but not fathers) of offspring with lower values of some testosterone-dependent traits (shoulder width, jaw width) had higher chances of becoming longevous. More generally—although most of the common causes of death can be behaviorally prevented or postponed (68–70), and individual behavior and success often correlate with testosterone-dependent facial and bodily traits (46, 71–73), the scarcity of associations between testosterone-dependent traits of offspring and longevity of parents was surprising.

### 4.2. Comparison with previous studies

#### 4.2.1. Height

Previous inter-generational studies of longevity have concentrated on the association between polygenic scores (PGS) of offspring traits and parental survival beyond the age corresponding to the 90th/99th survival percentile (18, 20, 23) or centenarians (33). Or results concentrating on offspring phenotypes are not directly comparable to the findings of the studies listed above, but some common patterns emerge. Height-increasing polygenic scores were inversely associated with extreme longevity in Japanese women but not men (33), which is similar to the associations between offspring height and parental longevity in our study. In UKBB, genetically predicted height was unrelated to parental longevity, while PGS for height at age 10 were negatively associated with longevity [[23; see also (31)]. These studies, however, did not stratify parental longevity by sex.

**Figure 4:**
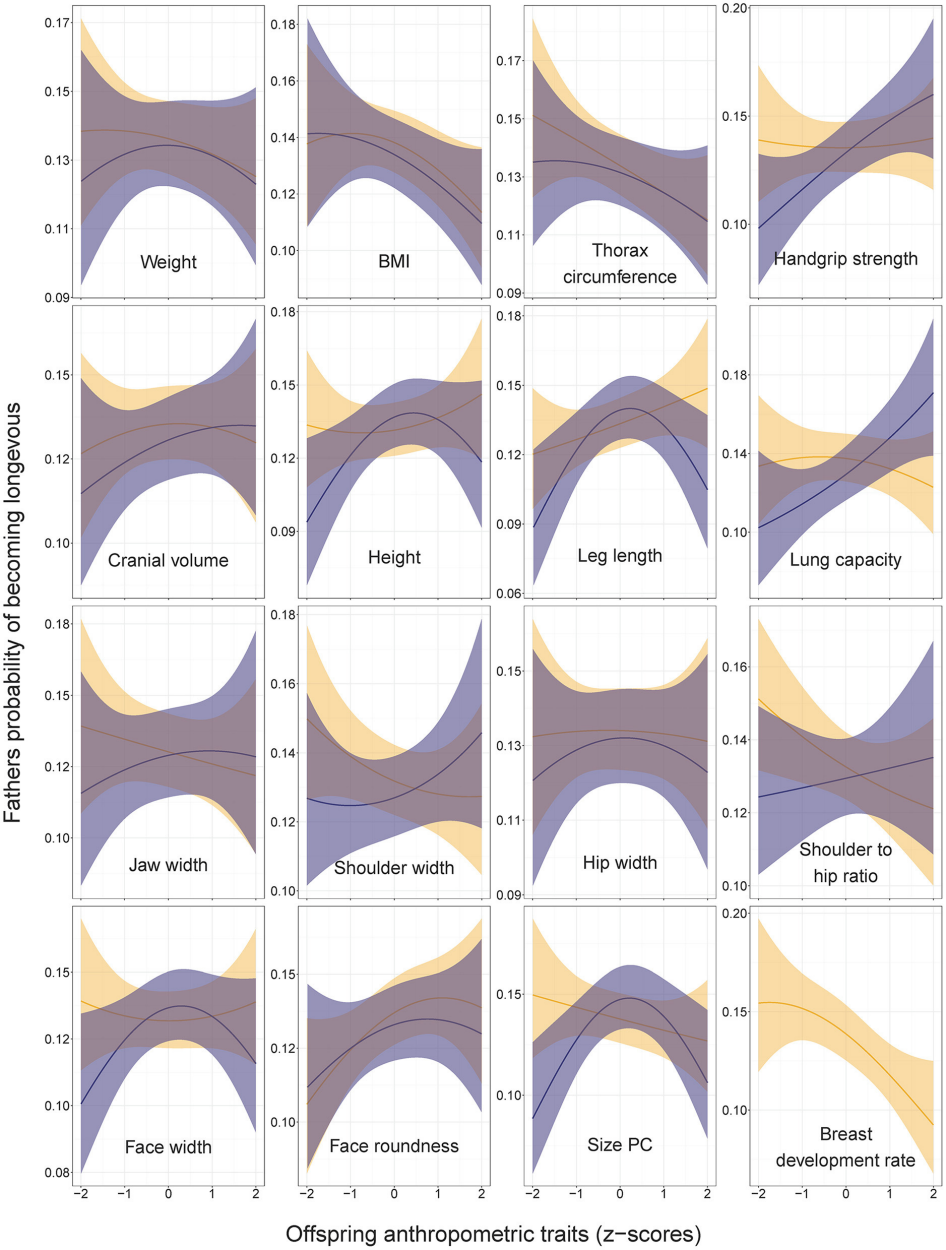
Fathers' probabilities of becoming longevous in relation to anthropometric traits of their sons (dark shading) and daughters (light shading). Predictions (with 95% CI) from the models without covariates: lrm[formula = longevity ~ focal_trait + (focal_trait*focal_trait)]. Statistics are provided in Supplementary Table 1 in ESM.

Our findings of associations between the height of children and parental longevity differ from those of the Swedish study of military conscripts, which showed that both mothers and fathers of taller sons had lower overall mortality (26). In our study, the son's height, leg length and size PC showed a bell-shaped relationship with fathers' longevity (Figure 3), while the longevity of mothers linearly decreased with the height (and most other linear measures) of their children irrespective of their sex (Figures 3, 4).

These findings contradict the common pattern of predominantly inverse (individual-level) associations of height and leg length with all-cause (and particularly cardiovascular and respiratory disease) mortality found in a large number of studies in developed countries (reviewed by 26, 74). Although height is often positively associated with many forms of non-smoking related cancers (74), it is doubtful that cancer mortality (that comprised only 22% of female deaths during our study period) could be a major reason for the negative association between offspring height and maternal longevity in our study. In this context, it is interesting that a study in UKBB found that genetic scores for the height of girls at age 10 and the height of boys at age 12 were positively correlated with parental mortality (*r*_g_ = 0.12) [(31); see also (75)]. Altogether these findings indicate that negative associations between height and mortality are not necessarily ubiquitous. However, we cannot also exclude the possibility that negative associations between mothers' longevity and height of their children primarily indicate that children of women with a propensity for long life just grow at slower rates, catching up with their peers at older ages.

#### 4.2.2. BMI

Our findings that mothers of children with high body mass and BMI had low chances of becoming longevous are perhaps most easily reconcilable with previous knowledge about associations between metabolism and cardiovascular health: Genetic propensity for obesity is associated with genes implicated in metabolic diseases (76) so that obese phenotype of children predicts shorter lifespan of their parents due to common genetic variants affecting both traits. In our study, the BMI of offspring was (irrespective of their sex) negatively associated with the longevity of their mothers, while in the case of fathers, the association was in the same direction but not formally significant (Figures 3, 4, Supplementary Table 1 in ESM). Studies of Swedish conscripts have found a positive association between the BMI of sons and the mortality of both their fathers and mothers (25, 29). Our findings contribute to previous knowledge by showing that not only sons' but also daughters' BMI predicts parental longevity. Our findings are also consistent with those of UKBB, showing that obesity of children correlated genetically with the mortality of their parents [*r*_g_ = 0.30 (31)].

#### 4.2.3. Other traits

As regards other anthropometric traits then, our findings that the lung capacity of sons was a relatively strong predictor of the longevity of their fathers (OR = 1.15, CI = 1.03–1.29) is partly consistent with findings of the study performed in Michigan during 1959–60 and showing that greater longevity (death after age 65) of either parent was associated with high values of ventilatory lung function among the sons (30). Interestingly, we found only father-son associations, as the lung capacity of daughters was not associated with parental longevity. Furthermore, sons' lung capacity was inversely associated with the longevity of their mothers (Figure 3). Handgrip strength of sons (but not daughters) predicted the longevity of their fathers but not mothers, while the longevity of mothers was independent of the strength of their children (Figures 3, 4).

Probably the most parsimonious explanation for the observed patterns is that the associations between paternal longevity and the robustness of their sons mainly stem from genetic causes. Because children share the same socio-economic and lifestyle confounders with both of their parents, we should have seen similar associations between offspring traits and longevity of both mothers and fathers in case if such associations were mainly caused by external environmental influences such as socially inherited SEP. Further, the inverse association between paternal longevity and daughters' lung capacity suggests that genetic associations between offspring phenotype and parental longevity are sex-specific. Regarding the longevity of mothers, sex-specific association emerged with respect to the cranial volume of their children: mothers of daughters (but not sons) with large heads had lower chances of becoming longevous (Figure 3). Sex-specific associations between offspring phenotype and parental longevity are consistent with findings of genealogical studies showing that father-son and mother-daughter inheritance patterns of lifespan are more common than father-daughter and mother-son inheritance patterns (15).

### 4.3. Conclusions, limitations, and implications

This study showed that children's anthropometric traits predicted their parents' longevity better in the case of mothers than fathers. Mothers of small-bodied children and fathers of vigorous sons had higher chances of becoming longevous. Some of our findings (such as negative associations between offspring weight and BMI vs. parental survival) were consistent with previous empirical and theoretical knowledge about processes affecting lifespan. Other findings were unexpected, such as inconsistent relationships between different testosterone-dependent traits and cranial volume of offspring vs. parental longevity. In particular, our findings failed to support the predictions that traits characteristic of to slow pace of life, such as high somatic investment into body and brain growth, cluster with long life.

One of the limitations of this study is the absence of information on the causes of parental deaths. For instance, the hypothesis that anthropometric traits that reflect testosterone exposure (and/or amount in circulation) associate with risk-prone behavior predicts that such testosterone-dependent traits associate specifically with mortality due to external causes in men. The absence of information about the causes of death of fathers prevented explicit testing of this hypothesis. Another limitation is the absence of information about possible sex-specific heritabilities of anthropometric traits and lifespan in the studied population. For instance, the sex-specific association between offspring traits and parental longevity could be explained if the inheritance of lifespan and/or morphometric traits would appear higher in the maternal than paternal line.

The central contribution of this study is that anthropometric traits of offspring were differently associated with mortality of their mothers and fathers and that some of these associations depended on the sex of offspring. This implies that future studies of longevity (including GWAS) would benefit from analyzing the associations between parental and offspring traits in a sex-specific manner. An important question arising from this study is whether or how much our findings are characteristic of a population with an extensive (9–10 years) sex difference in life expectancy. Future research would benefit from studies in societies with a smaller gender gap in lifespan to test our results' generalizability.

## Data availability statement

The raw data presented in this article is available in Supplementary material file Table 4. Original data cards are stored in Tartu Ülikooli museum and can be accessed here: https://www.muis.ee/en_GB/museaalview/3451136.

## Ethics statement

The studies involving human participants were reviewed and approved by Data processing was performed anonymously under the license of the Research Ethics Committee of the University of Tartu (protocol # 275/ T-1, issued on 20.11.2017) and approved by the Estonian Data Protection Directorate (Decision n2 2.2.-1/17/55, issued on 30.01.2018). Written informed consent for participation was not provided by the participants' legal guardians/next of kin because: The study was performed between 1956 and 1969, when Estonia was occupied by Soviet Union and written consent was not required for this kind of research at that time.

## Author contributions

MV located the data of schoolchildren, built up, and complemented the database. MV and PH designed the study and wrote the manuscript. RM performed the statistical analyses. All authors contributed to the article and approved the submitted version.
