# Supplementary material for: Mothers of small-bodied children and fathers of vigorous sons live longer
Source: Front Public Health. 2023 Jan 25;11:1057146. doi: 10.3389/fpubh.2023.1057146 (PMC9905732; doi:10.3389/fpubh.2023.1057146)
Supplement: Supplementary file 5 [file Table_5.docx]

Fig. S1. Dynamics of three major causes of death in Estonia from 1989-2018. Source: Estonian Health Statistics and Health Research Database, <https://statistika.tai.ee/index_en.html>

<https://statistika.tai.ee/pxweb/en/Andmebaas/Andmebaas__01Rahvastik__04Surmad/SD21.px/>


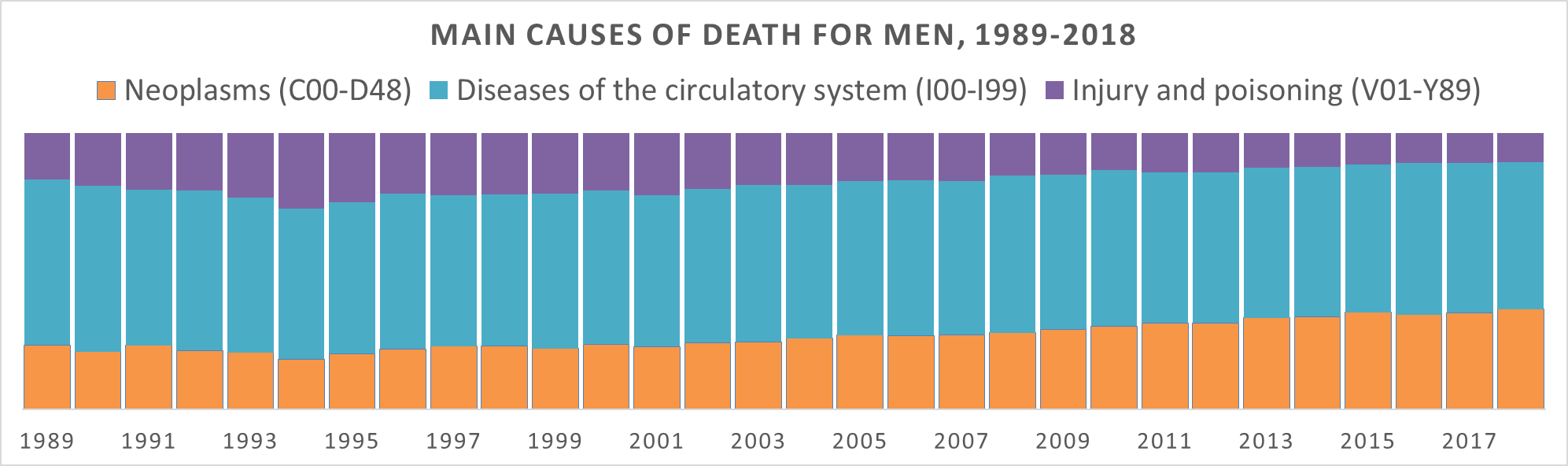


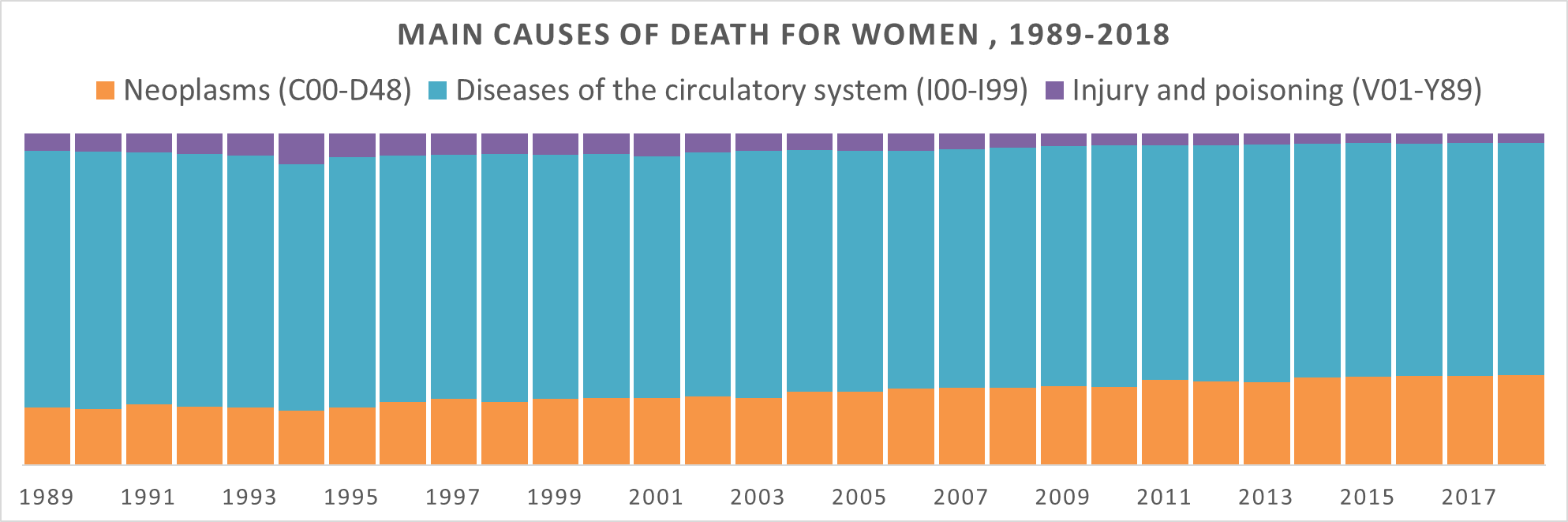


**Average: 1989 - 2018**

**Men Women**
